# Testing for personality consistency across naturally occurring behavioral contexts in sanctuary chimpanzees (*Pan troglodytes*)

**DOI:** 10.1002/ajp.23451

**Published:** 2022-11-17

**Authors:** Hélène Chotard, Kim A. Bard, Jérôme Micheletta, Marina Davila‐Ross

**Affiliations:** ^1^ Department of Psychology, Centre for Comparative and Evolutionary Psychology University of Portsmouth Portsmouth UK

**Keywords:** behavior‐based traits, contextual consistency, great apes, real‐life settings, temporal consistency

## Abstract

Personality is both a reflection of the bio‐behavioral profile of individuals and a summary of how they typically interact with their physical and social world. Personality is usually defined as having distinct behavioral characteristics, which are assumed to be consistent over time and across contexts. Like other mammals, primates have individual differences in personality. Although temporal consistency is sometimes measured in primates, and contextual consistency is sometimes measured across experimental contexts, it is rare to measure both in the same individuals and outside of experimental settings. Here, we aim to measure both temporal and contextual consistency in chimpanzees, assessing their personality with behavioral observations from naturally occurring contexts (i.e., real‐life settings). We measured personality‐based behaviors in 22 sanctuary chimpanzees, in the contexts of feeding, affiliation, resting, and solitude, across two time periods, spanning 4 years. Of the 22 behaviors recorded, about 64% were consistent across two to four contexts and 50% were consistent over time. Ten behaviors loaded significantly onto three trait components: explorativeness, boldness‐sociability, and anxiety‐sociability, as revealed by factor analysis. Like others, we documented individual differences in the personality of chimpanzees based on reliably measured observations in real‐life contexts. Furthermore, we demonstrated relatively strong, but not absolute, temporal, and contextual consistency in personality‐based behaviors. We also found another aspect of individual differences in personality, specifically, the extent to which individual chimpanzees show consistency. Some individuals showed contextual and temporal consistency, whereas others show significant variation across behaviors, contexts, and/or time. We speculate that the relative degree of consistency in personality may vary within chimpanzees. It may be that different primate species vary in the extent to which individuals show consistency of personality traits. Our behavioral‐based assessment can be used with wild populations, increasing the validity of personality studies, facilitating comparative studies and potentially being applicable to conservation efforts.

## Research Highlights

- Chimpanzees show consistency in their personality‐based behaviors across naturally occurring contexts and over time.
- Individuals show different contextual and temporal consistency of their personality‐based behavior profile.

## Graphical abstract

Sanctuary chimpanzees show consistency of their personality‐based behaviors across real‐life contexts and over time while displaying interindividual differences in the consistency of their personality‐based behavioral profile

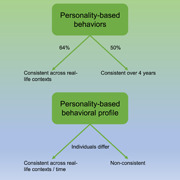

**Abbreviations**
A: affiliation
C: consistency
CWO: Chimfunshi Wildlife Orphanage
F: feeding
ICC: intraclass correlation coefficient
KMO: Kaiser–Meyer–Olkin
LM: linear model
NC: no consistency
R: resting
REFA: regularized factor analysis
S: solitude
VIF: variance inflation factors

## 1 INTRODUCTION

Personality has been defined as interindividual differences in behavior‐based traits (Allport, 1961; Funder, 2001). In humans, an individual's personality, in general, is expected to show consistency over time (Roberts & DelVecchio, 2000; Weisbuch et al., 2010) as well as consistency across different situations (Funder & Colvin, 1991; Furr & Funder, 2004; Moskowitz, 1982; Sherman et al., 2010; Weisbuch et al., 2010). Only a few personality researchers think that human personality fluctuates across contexts (e.g., Mischel, 1968; Mischel & Peake, 1982). Individuals differ in the way they express their personality traits; that is, their behaviors may fluctuate when interacting with both their physical and social environments in the natural course of their lives (Ickes et al., 1997; Mehl et al., 2006). Here, we add to our understanding of personality by considering both contextual consistency and temporal consistency in the personality of chimpanzees using naturalistic behavioral observations (hereafter behavioral observations). More specifically, we measure behaviors that index different personality traits (hereafter personality‐based behaviors) across a variety of distinct real‐life contexts (a context was defined here as a broad category regrouping multiple specific situations; Sih, Bell, Johnson, 2004), and over a 4‐year period.

As in human primates, consistency has been considered to be a key criterion in the personality of nonhuman primates (e.g., Brent et al., 2014; Massen et al., 2013; Weiss et al., 2007). Researchers consider that personality is consistent when individuals show stability in their personality‐based behaviors while maintaining their differences with other members of the group, that is, the rank‐order consistency (stability of the relative position of individuals on a personality trait over time and context; Roberts & DelVecchio, 2000). Personality consistency has been found using rating methods (e.g., Dutton, 2008; Uher & Asendorpf, 2008; Weiss & King, 2015), experiments (e.g., Koski & Burkart, 2015; Kutsukake et al., 2012; Massen et al., 2013) and to a smaller extent, via behavioral observations (e.g., Koski, 2011; Seyfarth et al., 2012; Tkaczynski et al., 2018). More specifically, temporal consistency has been found using all three methodologies (see Freeman & Gosling, 2010; Gosling, 2001); behavioral observations revealed temporal consistency for personality traits (e.g., sociability, boldness, anxiety, boldness) in tufted capuchin (*Sapajus apella*: Byrne & Suomi, 1995), five macaque species (*Macaca mulatta*: von Borell et al., 2016; *M. nemestrina:* Reite & Short, 1980 *; M. nigra*: Neumann et al., 2013; *M. silenus*: (Kluiver et al., 2022); *M. sylvanus*: Tkaczynski et al., 2018), chacma baboons (*Papio ursinus*: Seyfarth et al., 2012), and in chimpanzees (*Pan troglodytes*: Koski, 2011). In contrast, contextual consistency has been investigated only with the experimental method, but for a range of personality traits, such as sociability, boldness, explorativeness, and anxiety in chimpanzees (e.g., Kutsukake et al., 2012; Massen et al., 2013; Uher et al., 2008), and across different nonhuman primate species (Weiss et al., 2011). Contextual consistency has not yet been examined using behavioral observations.

So far, behavioral methods have been primarily used to validate or complement rating scales, or to supplement experimental measures in the assessment of personality consistency (e.g., Clay et al., 2015; Freeman et al., 2013; Pederson et al., 2005). It is important to note that these different methods to measure personality are likely to provide different information about a personality trait. Indeed, different methods may illuminate different facets of a particular trait (Carter et al., 2012). For example, Carter et (2012) assessed boldness in wild chacma baboons and found positive correlations between ratings and experimental observations for one of the two factors only. In this study, the rating approach highlighted boldness across multiple contexts (e.g., “the baboon retreats readily from others or outside disturbances”; “the baboon behaves in a positive, assured and bold manner, not restrained or tentative”), whereas the experimental observations were specific to one context (i.e., reactions to novel food). Although both methods provide a measure of boldness, the two methods may have captured slightly different aspects of the boldness personality trait.

There are strengths and weaknesses of different methods used in animal personality research (Freeman et al., 2011). Ratings allow researchers to obtain personality data quickly, in large samples, and in various settings (e.g., Tkaczynski et al., 2018) which are important advantages, but are often criticized for their subjectivity and anthropomorphism (Uher, 2008). The experimental method is standardized and controlled (Freeman et al., 2011) but often focuses on a limited number of behaviors displayed in specific situations (e.g., Uher et al., 2008). Moreover, as our example above illustrates, experimental observations often are limited both to a specific facet of a personality trait and to a particular trait (e.g., boldness to novelty in a food context: Carter et al., 2012). Although a behavioral approach can be time‐consuming in terms of data collection and coding (Freeman et al., 2011), and possibly introduce large variability when the data set is small, this method offers important benefits. The benefits of a behavioral approach include high ecological validity, with observations covering a range of distinct contexts (e.g., play, vigilance, feeding) and behaviors assessing multiple personality traits, which can be sampled over multiple time periods. These advantages of the behavioral approach contribute to a more detailed description of a fuller range of an individual's personality, and a description of personality that is tied to the contexts in which individuals live and behave (Mehta & Gosling, 2008; Uher, 2008); for example, the trait of sociability can be observed in a food sharing context (Silk et al., 2013) and in social proximity, while grooming, resting, or locomoting (Massen & Koski, 2014). Primate studies using the behavioral approach are needed because, to date, none have systematically assessed contextual consistency, even though several personality studies have used behavioral observations to measure temporal consistency (e.g., Brent et al., 2014; Capitanio, 1999; Kluiver et al., 2022; Konečná et al., 2008, 2012; Koski, 2011; Neumann et al., 2013; Pederson et al., 2005; Schaefer & Steklis, 2014; Seyfarth et al., 2012). Personality is defined as an aspect of an individual that is consistent across contexts and is consistent over time (Gosling, 2008; Sih, Bell, Johnson, & Ziemba, 2004). Thus, we need to have an approach that can assess both temporal and contextual consistency. With an observational approach, we can understand better how the different behaviors that constitute personality traits work together. The factor of highest importance is to have data that address how personality‐based behaviors are expressed in the daily life of individuals.

Regardless of the method of assessment used (rating, experimental, or behavioral observations) and the research field (Carter et al., 2013), the label of a personality trait can be described in different ways while still sharing the same meaning. In chimpanzees, past research using ratings described their personality by six dimensions—dominance, extraversion, conscientiousness, agreeableness, neuroticism, and openness (e.g., King & Figueredo, 1997; Pederson et al., 2005). Other experimental and behavioral studies described, instead, these traits differently (Réale et al., 2007) such as exploration tendency as an equivalent of openness, or sociability as an analog name for extraversion and agreeableness (Koski, 2011; Massen et al., 2013; Uher et al., 2008). Whereas raters are typically asked how much a trait label reflects an individual's personality, observational methods rely on documenting the actual occurrences of different trait labels. Thus, a behavioral method has the advantage of being bottom‐up, which means that the trait labels derive only from the behaviors which are chosen and coded by the researchers.

This study used a bottom‐up approach that targeted behaviors that had previously been associated with four personality traits: sociability, boldness, explorativeness, and anxiety. By using such an approach, we wanted to capture a broad picture of these four traits and develop a coding scheme that relied on the review of a wide range of studies in nonhuman primates, including personality and behavioral research in which personality assessment was not necessarily the main objective. These four traits are behavior‐based and have been shown to be reliable in ratings (Clay et al., 2015; Freeman et al., 2013), experimental observations (Massen et al., 2013; Uher, Asendorpf & Call, 2008) and behavioral observations (Koski, 2011; Uher et al., 2008). Sociability is defined as closeness to others, is demonstrated through social preferences, and can be observed for instance, in social proximity or grooming (Eckardt et al., 2015; Koski, 2011; Pederson et al., 2005). Boldness is the willingness to engage or place themselves in risky, potentially harmful situations and can be observed through actions such as hitting, chasing, or approaching under conditions in which the outcomes are unknown (Anestis, 2005; Massen et al., 2013; Nishida et al., 1999). Explorativeness, defined as showing a keen interest in objects or conspecifics, can be observed in forms of gazing, combined with actions, such as touching, approaching, and manipulating (Forss et al., 2015; Kutsukake et al., 2012; Schuppli et al., 2017). Finally, anxiety, evident in fearful, stressful, or tense responses towards potential dangers, can be measurable through the observation of self‐directed or vigilant behaviors (Baker & Aureli, 1997; Kutsukake et al., 2012; Uher et al., 2008). These traits are ecologically and evolutionary relevant (Réale et al., 2007; Smith & Blumstein, 2008) as they are argued to affect the fitness of individuals (e.g., life span: Altschul et al., 2018; survival: Silk et al., 2003). In addition, the expression of these personality traits is widespread across different primate species (Uher, 2008).

In the current study, we tested whether the personality‐based behaviors of 22 sanctuary chimpanzees were consistent across ecologically relevant life contexts in a nonexperimental study and setting. Based on previous experimental studies in captive chimpanzees (e.g., Kutsukake et al., 2012; Massen et al., 2013; Uher et al., 2008), we hypothesized that the expression of the personality‐based behaviors would be consistent across distinct naturally occurring contexts. In addition, based on previous research in chimpanzees (Koski, 2011; Uher et al., 2008), we hypothesized that the personality‐based behaviors would show temporal consistency across two time periods (4 years apart).

## 2 METHODS

This study involved only noninvasive behavioral observations of the individuals. The study complied with protocols approved by the University of Portsmouth's Animal Welfare and Ethical Review Body, as well as Chimfunshi Research Advisory Board. All methods for this study adhered to the legal requirements of Zambia and the American Society of Primatologists' Principles for the Ethical Treatment of Nonhuman Primates.

### 2.1 Subjects and study site

Twenty‐two chimpanzees (8 females) living at Chimfunshi Wildlife Orphanage (Zambia) were included in this study, with an age range of 5 and 32 years (mean ± SD: 16 ± 9.62) (see Supplementary Information: Table S1). The subjects were members of two stable, multi‐male multi‐female colonies, of 11 and 25 chimpanzees showing natural fission–fusion dynamics, and lived in enclosures of 25 and 77 ha, respectively. The sex and age ratios of the groups differed slightly between 2013 and 2017 as a result of the deaths and births within each colony (Enclosure 1: 10 males and 14 females in 2013, and 9 males and 16 females in 2017; Enclosure 4: 8 males and 3 females in 2013, and 9 males and 2 females in 2017).

Each enclosure contained native fruit groves, grasslands, and forests in the Miombo woodland, as well as an indoor area (used for midday feeding or medical check‐up). The chimpanzees were provisioned with food (e.g., local fruits and vegetables) inside around noon and outside in the afternoon each day, and they could also forage in the forest. Water was always available outside via a water fountain.

The initial formation of the colonies took 2–5 years and the last re‐structuring of the colony ended at least 11 years before data collection. The two colonies were composed of a mix of wild‐born chimpanzees and chimpanzees born at CWO. Most of the chimpanzees born at CWO were mother‐reared; some were temporarily removed for health checks but were reunited with their mother as soon as all health concerns were treated. The wild‐born chimpanzees had different background experiences, including varying amounts of time with their mothers before various experiences with humans (e.g., pet trade) and placement in the sanctuary. The initial colonies were formed by arrival dates of the wild‐born chimpanzees rather than their geographic background. The wild‐born chimpanzees were brought to CWO, individually or in pairs, from countries where wild chimpanzees live (e.g., Tanzania, Uganda, and Rwanda). If they were born in the countries they were sent from, then the subspecies representation for these individuals would be 42%–65% for *Pan troglodytes schweinfurthii* and 31%–42% for *P. t. troglodytes* (Maisels et al., 2016; Plumptre et al., 2016). Although we do not know with certainty, the colonies are likely composed of a comparable mix of sub‐species, with no apparent phylogenetic differences (Rawlings et al., 2013).

### 2.2 Data collection

Video‐recordings were collected using focal animal sampling (Altmann, 1974) over two field seasons, between June and September 2013 and between May and September 2017. The subjects were followed for 4 min (2013) or 5 min (2017), once or twice a day during the morning (from 7:30 to 11:30 a.m.) and/or afternoon (from 1 to 5:30 p.m.) sessions. Before each recording session, the order of focal animal sampling was randomized to avoid any bias towards the same individual and provide a balance between time periods and contexts. Thus, all subjects were observed at different times throughout the day and in different contexts (social and nonsocial: see below for more details). Each conspecific was individually identified by the observer as soon as they were within 10 m of the focal subject. In total, 53 h of recording were used for this study, with approximately 2.5 h per individual (mean ± SD = 2.43 ± 0.50 h), collected in approximately 32 focal observations per individual (31.45 ± 7.56 focal).

### 2.3 Behavioral coding

We designed a very detailed coding scheme to apply to the video‐recordings focusing on behaviors and context. Overall, we coded 22 behaviors and 8 contexts.

Our choice of the 22 behaviors was based on previous personality and behavioral studies in nonhuman primates to reflect four personality traits: sociability, boldness, explorativeness, and anxiety (see Supporting Information: Table S2). To capture as much diversity of expression of these personality traits as possible, we also selected some behaviors that might not be always strong indicators of the personality trait of interest (e.g., food sharing representing sociability). Supporting Information: Table S2 depicts examples of previous personality and behavioral studies on nonhuman primates mentioning the 22 measured behaviors.

By using video‐recordings, we were able to code the behavioral actions independently from the contexts, that is, the recordings were played, first, to code the behavioral actions, and then, were played separately to identify the contexts. An individual action was not considered part of a series of actions if it was separated in time by at least 5 s from other actions. This approach allowed us to ensure the independence of the occurrences and avoid a possible inflated estimation of interindividual differences, allowing us, hence, to capture information as precisely as possible. For each type of behavior, we computed frequency per hour of context. Each subject obtained one score per behavior for each context, and each score was standardized as a *z*‐score based on all chimpanzees' scores on a specific behavior within a specific context.

In experimental studies, a context is determined by the presence of an object or experimental device placed in the environment surrounding a subject. For instance, a snake can be hidden in the enclosure which, then, corresponds to an encounter with a predator (Koski & Burkart, 2015; Massen et al., 2013; Šlipogor et al., 2016). Here, we used a similar approach but instead of the object, we considered the conspecific(s) as the key cue to define the context. More specifically, the context was coded with details and defined according to the presence and activity of the conspecifics (not of the subject) present within 10  m of the focal. To be considered, a context had to be displayed by at least half of the conspecifics surrounding the focal subject and last at least 10 s; it started from the first behavioral indicator defining the context displayed by the conspecifics. Due to their short occurrences, the duration requirement was relaxed to any length of time for three of the contexts (play, aggression, and vigilance; see Supporting Information: Table S3 for an overview of the mean duration of each context).

We initially classified the observations into eight naturally occurring contexts: feeding, play, grooming, resting, solitude, vigilance, aggression, and locomotion contexts (see Supporting Information: Table S3). However, only five occurred frequently enough to conduct meaningful analyses (see Supporting Information: Table S4). We combined grooming and play to create an affiliation context as they together represented approximately 10% of the overall data set (see Supporting Information: Table S4 for details). This balanced the durations of the four primary contexts, and was in line with previous personality studies (Kuhar et al., 2006; Pederson et al., 2005).

The data were coded by H.C. Another observer who was naïve about the hypotheses of the study coded 20% of the complete data set for reliability by measuring the behaviors independently from the contexts. The two coders reliably classified the same contexts within 3 s (i.e., a margin of error), Cohen's kappa (*κ* = 0.79). The two coders agreed on the type and number of behaviors, intraclass correlation coefficient (ICC) (mean  ICC (3,1) = 0.59, SD = 0.24, *p* < 0.05).

### 2.4 Statistical analysis

In the following section, we describe our analytic strategies to assess contextual and temporal consistency. Then, we describe how we established the personality structure of the chimpanzees in our study. As is usually done in primate personality research (e.g., Massen et al., 2013; Šlipogor et al., 2016), only the behaviors showing contextual and/or temporal consistency were subjected to a factor analysis. All analyses were computed using SPSS Statistics 25 (IBM).

#### 2.4.1 Testing for consistency

For the consistency analyses, we included all contexts that had a total duration across the two time periods that was at least 10% of the overall total duration of the data set (see Supporting Information: Table S4). This resulted in analyses of four contexts (feeding, affiliation, resting, solitude) allowing us to obtain a broad picture of the consistency of the subjects' personality‐based behaviors.

First, we used Cronbach's alpha to measure cross‐context consistency for each behavior. This measure allowed us to assess internal consistency, which described the extent to which a behavior is expressed similarly across different contexts (Bland & Altman, 1997; Cronbach, 1951). By using this method, we were able to obtain a more detailed understanding of how consistent the behaviors were, and thus determine whether the level of expression of some behaviors was higher in some contexts compared to others. Cronbach's alpha is computed from the number of contexts that are included in the study, the average inter‐context covariance among the contexts, and the average variance. The value increases if the number of contexts or the average inter‐item correlation increases. We choose to consider any value above 0.6 as indicating good consistency across contexts (Hair et al., 2014; Peterson, 1994). Here, if the alpha value was lower than 0.6, a stepwise approach was used to re‐estimate the Cronbach's alpha value, with the removal of a less relevant context. With this approach, we found the limit of cross‐context consistency and identified the strength of consistency between contexts for each behavior by dropping the less relevant contexts one at a time (Field, 2013). In other words, this analysis reported the consistency of behaviors across contexts, starting with assessing consistency in all four contexts and ending with a decision about whether a behavior was consistent across three or two contexts.

Second, we measured the temporal consistency of each behavior with the ICC using a two‐way mixed model— ICC(3,1)—with the time period as the fixed variable and individual identity as the random variable. ICC measures the proportion of total variance in behavior‐based traits that was due to differences between subjects while controlling the within‐subject variance (McGraw & Wong, 1996; Shrout & Fleiss, 1979). In behavioral studies, the two‐way mixed ICC is often used to assess the temporal consistency of variables (e.g., Koski, 2011; Massen et al., 2013). A behavior was considered consistent over time if the ICC value was significantly different from 0 (*p* < 0.05).

In addition, both contextual and temporal consistency were analyzed in two ways: variable‐centered and individual‐centered analyses (Furr & Funder, 2004; Uher et al., 2008). Indeed, the variable‐centered approach allowed us to characterize the consistency of individual differences in each behavior. In other words, we assessed the consistency of behaviors across contexts and across time. With the individual‐centered approach, we characterized the consistency of the overall behavioral profile of each individual. In other words, we assessed the consistency of an individual's personality across contexts and time.

#### 2.4.2 Determining the personality structure

We determined the personality structure of the chimpanzees of this study by doing a factor analysis with all the reliable behaviors from all four contexts (feeding, affiliation, resting, and solitude). Due to the small sample size, the behaviors were subjected to a regularized factor analysis (REFA: Jung & Lee, 2011), specifying unweighted least squares. To determine the number of factors to extract, we interpreted the scree‐plot and used the parallel analysis (Horn, 1965; O'Connor, 2000). Parallel analysis allowed to compare the eigenvalues obtained from the observed data set to the eigenvalues obtained from a random generation. This analysis uses the 95th percentile values from the random data set to determine how many factors are to be extracted; thus, requiring the percentile values to be below the corresponding eigenvalues from the observed data. Additionally, we used an orthogonal rotation (Varimax) for the REFA, and considered all behavior loadings >0.4 to be salient (Konečná et al., 2012; Tkaczynski et al., 2018). To determine which type of rotation was the most appropriate, the analysis was repeated by testing an oblique rotation (direct Oblimin) which allowed the factors to correlate. The correlations between the factors were relatively low and ranged between −0.06 and 0.05, suggesting the factors were independent. Additionally, the two rotations provided identical solutions regarding the behavior loadings. Therefore, we retained and interpreted Varimax‐rotated factors. Subsequently, all behaviors that loaded onto the same factor (i.e., personality trait) were summed to create personality factor scores for each subject, and were, then, standardized as *z*‐scores.

#### 2.4.3 Comparing personality scores depending on sex and age

We fitted linear models (LMs) using R (R Core Team, 2022) to assess the influence of sex (*N*_males_ = 14, *N*_females_ = 8), and age (continuous variable, range 7–34 years; based on median age 2013–2017) on the personality traits (response variable). Separate models were run for each personality trait. Sex, age, and enclosure (*N*_Enclosure 1_ = 13, *N*_Enclosure 4_ = 9) were included as fixed effects; the level of significance was set at 0.05. We checked for collinearity between the predictors using variance inflation factors (VIF, calculated with the vif function of the package car; Fox & Weisberg, 2010). The VIF for all variables ranged from 1.12 to 1.20, indicating that there was low collinearity; all variables were retained in the analysis (Zuur et al., 2009). The significance of the full model was tested by comparing it to a null model, which was an intercept only model. We checked the homoscedasticity and the normality of the residuals using the functions “plot” (package “base” v.4.2.1); all assumptions were met here.

## 3 RESULTS

Chimpanzees spent 90% of their time in one of four contexts: feeding, affiliation, resting, and solitude (Supporting Information: Table S4). Chimpanzees spent the least amount of time in the context of Aggression (0.2% in 2013 and 0.5% in 2017 of the total duration of the data set). The most time was spent in feeding in 2017 (46.3% of the total duration of the data set) and in resting in 2013 (36.3% of the total duration of the data set). The average duration of bouts for each context per chimpanzee also revealed four primary contexts (2.79 min per bout per chimpanzee in feeding, 2.69 min per bout per chimpanzee in affiliation, 1.78 min per bout per chimpanzee in resting, and 2.61 min per bout per chimpanzee in solitude: Supporting Information: Table S3).

**Figure 1:**
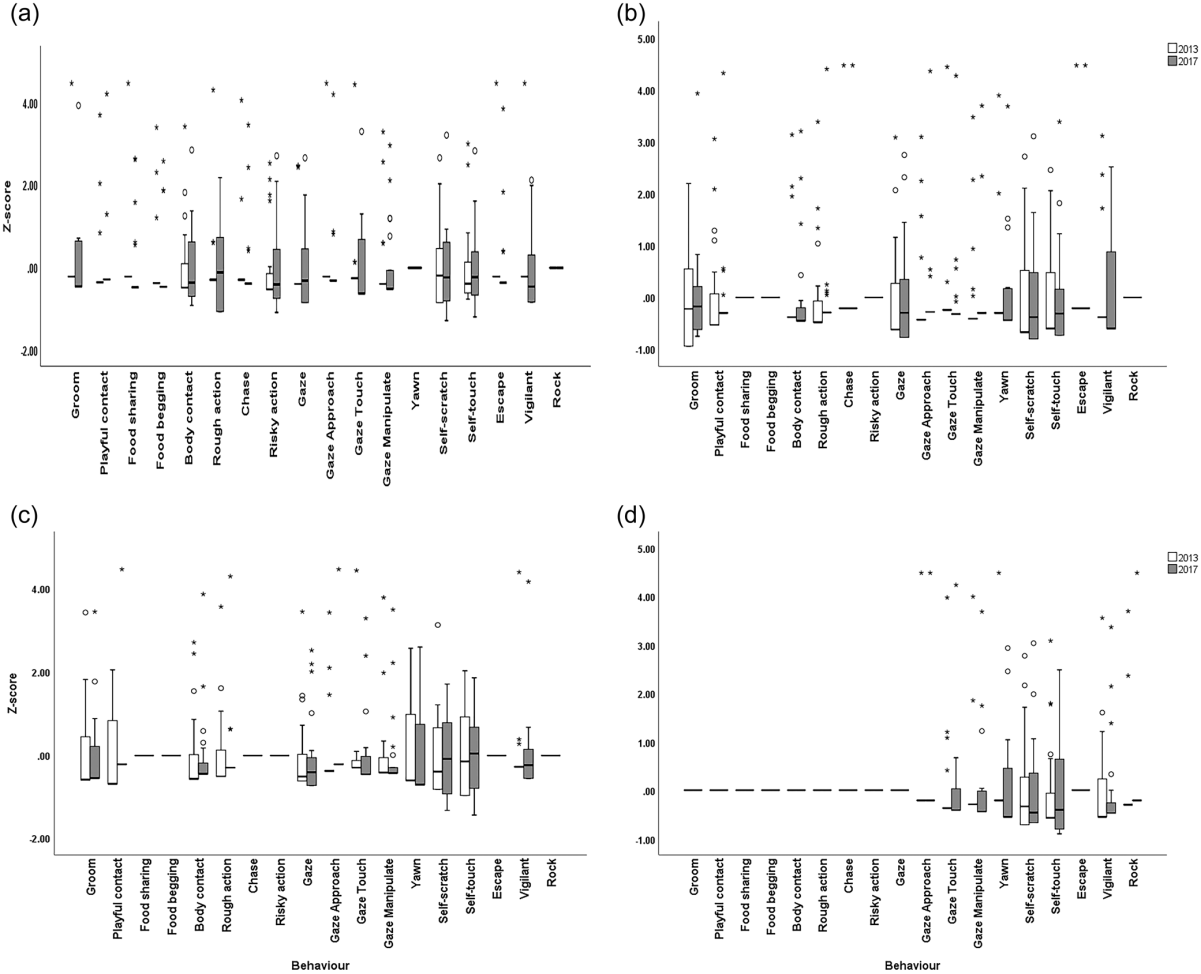
Visual representation of the behavioral expression across subjects per time period during (a) feeding, (b) affiliation, (c) resting, and (d) solitude contexts. Data are *z*‐scored within each time period (2013 and 2017). The thick horizontal lines indicate medians; the vertical length of the boxes corresponds to interquartile range; the thin short horizontal lines indicate the minimum and maximum values.

From the 22 behaviors measured, four behaviors were discarded from the analyses as they were observed only in one time period (Display, Risky approach, Pilo‐erection) or they did not occur at all (Throw). Thus, 18 behaviors were retained for further analyses (see Figure 1).

### 3.1 Contextual consistency

#### 3.1.1 Variable‐centered approach

To test for contextual consistency with the variable‐centered approach, the behavioral observations were combined over the two time periods. Here, 4 out of 18 behaviors were not retained for the contextual analysis as they were only expressed in one context (Food begging, Food sharing, Risky action, Rock). The analysis revealed that 64% of the behaviors showed acceptable Cronbach's alpha values across two to four contexts, suggesting that the behaviors differed in contextual consistency (Table 1). Five behaviors did not meet the criteria of contextual consistency (Cronbach's *α* < 0.60) when considering only the contexts in which they were displayed (Table 1). Supporting Information: Figure S1 shows an example of a low (Gaze Approach) and high (Gaze Touch) level of contextual consistency.

**Table 1:** Contextual consistency of the behaviors across feeding (F), affiliation (A), resting (R), and solitude (S) contexts

Contexts	Behaviors	Cronbach's *α*
F‐A‐R	Body contact	**0.69**
F‐A‐R	Groom	**0.73**
F‐A‐R	Playful contact	**0.60**
F‐A	Chase	**0.94**
F‐A‐R	Rough action	0.49
F‐A‐R	Gaze	−0.24
A‐R		0.19
F‐A‐R‐S	Gaze Approach	−0.26
A‐R‐S		0.16
A‐S		0.31
F‐A‐R‐S	Gaze Manipulate	**0.82**
F‐A‐R‐S	Gaze Touch	**0.68**
F‐A	Escape	0.35
F‐A‐R‐S	Self‐scratch	**0.70**
F‐A‐R‐S	Self‐touch	0.59
F‐A‐S		**0.67**
F‐A‐R‐S	Vigilant	**0.67**
A‐R‐S	Yawn	**0.69**

*Note*: All acceptable consistencies are in boldface.

An additional analysis was conducted when considering only the two most predominant contexts (feeding and resting) as they represented more than 25% of the overall data set in terms of duration (see Supporting Information:  Results and Tables S8, S9, S10).

#### 3.1.2 Individual‐centered approach

When assessing contextual consistency with the individual‐centered approach, some behaviors were not retained for the analysis. Here, only 6 out of 18 behaviors were part of the behavioral profile of the individuals as we included only behaviors that were expressed in all four contexts. We found that 4 of 22 individuals (18% of the sample) showed contextual consistency in their behavioral profiles. The other 18 individuals did not show context consistency in their behavioral profiles (see Table 2).

**Table 2:** Individual‐centered analysis of contextual and temporal consistency when considering all four contexts

	Context	Time	95% confidence interval		*F* value	*p* Value
ID	Cronbach's *α*	ICC(3,1)	Lower bound	Upper bound		
1	0.36	0.11	−0.37	0.54	1.24	0.331
3	0.42	−0.04	−0.49	0.42	0.93	0.562
4	−0.66	0.09	−0.38	0.53	1.20	0.353
7	−0.16	−0.09	−0.52	0.38	0.84	0.636
8	0.18	0.29	−0.20	0.66	1.80	0.118
10	−0.99	0.06	−0.41	0.50	1.13	0.401
13	0.45	0.05	−0.42	0.49	1.10	0.426
15	0.04	0.21	−0.27	0.61	1.54	0.190
16	0.18	0.16	−0.32	0.57	1.38	0.259
24	−0.05	0.36	−0.12	0.70	2.11	0.066
29	**0.71**	**0.58**	0.16	0.82	3.70	0.005
32	**0.75**	0.26	−0.23	0.64	1.69	0.145
36	−0.44	−0.03	−0.48	0.43	0.95	0.545
42	−0.27	−0.06	−0.50	0.41	0.89	0.597
47	0.55	0.18	−0.30	0.59	1.43	0.234
50	0.52	**0.56**	0.14	0.81	3.52	0.007
53	0.24	−0.03	−0.48	0.43	0.93	0.555
56	0.13	−0.12	−0.55	0.35	0.78	0.691
57	**0.61**	0.13	−0.34	0.55	1.31	0.294
60	0.02	**0.69**	0.33	0.87	5.35	0.001
65	0.38	0.28	−0.20	0.65	1.77	0.124
66	**0.94**	0.10	−0.37	0.53	1.23	0.338

*Note*: The behavioral profile of each individual included six behaviors (Gaze Approach, Gaze Touch, Gaze Manipulate, Self‐scratch, Self‐touch, Vigilant) and 18 behaviors (Groom, Playful contact, Food sharing, Food begging, Body contact, Rough action, Chase, Risky action, Gaze, Gaze Approach, Gaze Touch, Gaze Manipulate, Yawn, Self‐scratch, Self‐touch, Escape, Vigilant, Rock) for the contextual and temporal consistency analyses, respectively. All acceptable consistencies are in boldface

We decided to re‐do this analysis by considering only the social contexts (feeding, affiliation, and resting). This allowed for the addition of more diversity in the behavioral profile of the individuals by including more behaviors (i.e., 11 behaviors against 6 in the above analysis), which were expressed in all three social contexts. In this analysis, 6 of 22 individuals (27% of the sample) showed contextual consistency in their behavioral profiles (Supporting Information: Table S5).

### 3.2 Temporal consistency

#### 3.2.1 Variable‐centered approach

We assessed 18 behaviors for temporal consistency, combining behaviors across contexts in which they occurred. For instance, as body contact was displayed in three contexts of feeding, affiliation, and resting, only these contexts were combined. Fifty percent of the behaviors were considered consistent over time with ICCs significantly different from 0 (Table 3). We found variation in ICC values, which ranged from −0.16 to 0.96, indicating different patterns of temporal consistency across the behaviors. See Supporting Information: Figure S1 for an example of a low (Gaze Approach) and high (Gaze Touch) level of temporal consistency.

**Table 3:** Temporal consistency of the behaviors

Behaviors	ICC(3,1)	95% confidence interval		*F* value	*p* Value
		Lower bound	Upper bound		
Body contact	**0.45**	0.05	0.73	2.66	0.015
Groom	0.24	−0.20	0.59	1.62	0.140
Playful contact	**0.54**	0.16	0.78	3.30	0.004
Chase	**0.96**	0.91	0.98	53.68	0.000
Rough action	0.10	−0.33	0.49	1.21	0.332
Gaze	0.20	−0.23	0.57	1.51	0.177
Gaze Approach	−0.16	−0.54	0.27	0.72	0.771
Gaze Manipulate	**0.58**	0.23	0.80	3.81	0.002
Gaze Touch	**0.84**	0.65	0.93	11.46	0.000
Escape	−0.08	−0.48	0.35	0.85	0.640
Self‐scratch	**0.48**	0.08	0.75	2.85	0.010
Self‐touch	0.19	−0.25	0.56	1.45	0.199
Vigilant	**0.46**	0.06	0.74	2.73	0.013
Yawn	**0.55**	0.18	0.79	3.45	0.003
Rock	−0.07	−0.47	0.36	0.87	0.620
Food begging	**0.54**	0.17	0.78	3.37	0.004
Food sharing	−0.11	−0.50	0.32	0.81	0.685
Risky action	−0.05	−0.46	0.37	0.90	0.597

*Note*: All acceptable consistencies are in boldface.

As for the contextual analysis using the variable‐centered approach, we decided to conduct an additional temporal consistency analysis when considering only the two most predominant contexts (feeding and resting; see Supporting Information:  Results and Tables S8–S10).

#### 3.2.2 Individual‐centered approach

When assessing temporal consistency using the individual‐centered analysis, all 18 behaviors were part of the behavioral profile of the individuals. The analysis revealed temporal consistency in the behavioral profile for three individuals (ICC range: 0.56–0.69; *p* < 0.05) (14% of the sample); the other 19 individuals did not show time consistency in their behavioral profile between 2013 and 2017 (see Table 2).

As for the contextual analysis using the individual‐centered approach, we decided to re‐do this analysis to see how consistent individuals were when considering only social contexts (feeding, affiliation, and resting) as opposed to all contexts. Here, the behavioral profile of the individuals included 17 behaviors; 3 of 22 individuals (14% of the sample) showed temporal consistency in their behavioral profiles (Supporting Information: Table S5).

### 3.3 Consistency as an individual difference

**Figure 2:**
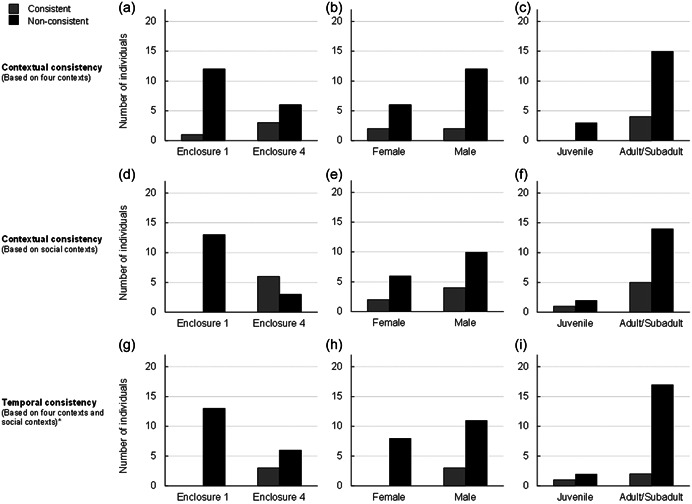
Visual representation of the number of individuals showing consistency in their behavioral profile within enclosures, sexes and age classes across four contexts (a, b, c, respectively), social contexts (d, e, f, respectively), and time (g, h, i, respectively). *Whether all four contexts or only social contexts were considered when assessing temporal consistency, there were always three individuals showing consistency; only the identity of the three individuals differed.

Eighteen chimpanzees did not show contextual consistency in their behavioral profiles across the four contexts. Among the four individuals who showed contextual consistency, three were from the same enclosure, two were females, and all were adults/subadults (see Figure 2a–c). Six chimpanzees showed contextual consistency when only the social contexts were considered. All were from the same enclosure, five were adult/subadult (three were male), and the juvenile was male (see Figure 2d–f).

Three individuals showed temporal consistency in their behavioral profiles when considering all four contexts or when considering only the social contexts (two of them showed temporal consistency in both analyses; see Supporting Information: Table S6). All were from the same enclosure, all were male, and two were adult/subadult (see Figure 2g–i; Supporting Information: Table S6). Interestingly, three also showed contextual consistency (in either the 4‐context analysis, the 3‐context analysis, or both). We did not run any statistical analyses of enclosure, sex, or age group, due to the small sample size.

### 3.4 Personality structure

We conducted an REFA to determine the structure of the personality of the chimpanzees in our study. Here, we decided to include all behaviors that showed consistency of at least one type (across contexts but not time, over time but not contexts, or over both context and time) to make use of all available data; hence, they were averaged accordingly. For instance, as Groom did not show significant temporal consistency, we included Groom in 2013 and Groom in 2017 as two separate behaviors in the REFA. A total of 11 behaviors were retained for the REFA: Body contact, Groom in 2013, Groom in 2017, Playful contact, Food begging, Chase, Gaze Manipulate, Gaze Touch, Self‐scratch, Vigilant and Yawn.

With these 11 behaviors, Kaiser–Meyer–Olkin (KMO = 0.498) was not acceptable, indicating poor suitability of the data (Budaev, 2010), although Bartlett's Test of Sphericity (*p* < 0.001) was acceptable. Consequently, we removed one behavior (Food begging) from the sample based on its individual KMO which was low (KMO < 0.5), suggesting its unsuitability for the analysis (Field, 2013). Therefore, 10 behaviors were retained for the final REFA. The Kaiser–Meyer–Olkin (KMO = 0.528) and Bartlett's Test of Sphericity (*p* < 0.001) indicated the suitability of the data.

Based on the scree‐plot and the parallel analysis, we extracted three factors that explained 58.6% of the variance (see Table 4). The first factor explained 23.4% of the variance and had positive loadings of two behaviors that were related to explorative actions; this factor was labeled “explorativeness.” The second factor explained 18.6% of the variance and had positive loadings of one behavior related to bold actions and two behaviors that related to social behaviors; this factor was labeled “boldness‐sociability.” Finally, the third factor accounted for 16.6% of the variance and had positive loadings of three behaviors that related to anxious actions and one behavior that was related to social actions; we labeled this factor “anxiety‐sociability.” One behavior did not load on any of the three factors: Groom displayed in 2017.

**Table 4:** Behavior loadings after Varimax rotation when considering feeding, affiliation, resting, and solitude contexts

Behavior	Explorativeness	Boldness‐sociability	Anxiety‐sociability
Gaze Touch	**0.997**	−0.064	0.006
Gaze Manipulate	**0.907**	0.187	−0.051
Chase	0.117	**0.886**	−0.096
Playful contact	0.077	**0.762**	−0.128
Body contact	−0.114	**0.702**	(0.488)
Groom in 2013	0.068	−0.053	**0.645**
Vigilant	−0.075	0.016	**0.630**
Self‐scratch	(0.411)	0.219	**0.590**
Yawn	0.027	−0.201	**0.484**
Groom in 2017	−0.202	0.080	0.359

*Note*: Loadings above |0.4| are in boldface. Behaviors that loaded significantly on more than one factor are in brackets.

### 3.5 Personality scores with sex and age comparisons

Based on the REFA solution, the behaviors that loaded onto the same factor were summed to create individual scores per subject for each of the three personality traits. The personality scores were, then, standardized as *z*‐score. The full‐null model comparison revealed that the test predictors (i.e., sex, age, enclosure) did not have a significant effect on explorativeness (*χ* ^2^ = 0.99, df = 3, *p* = 0.804) and boldness‐sociability (*χ* ^2^ = 4.68, df = 3, *p* = 0.197); therefore, we did not consider predictors of these two models further. However, enclosure significantly predicted the score of anxiety‐sociability (*β* = 1.66, *p* < 0.001; see Supporting Information: Table S7), with individuals from Enclosure 4 scoring higher (mean ± SD = 0.87 ± 0.93) than individuals from Enclosure 1 (−0.60 ± 0.46).

## 4 DISCUSSION

Our study investigated consistency across varying contexts and across time with the observation of personality‐based behaviors in real‐life, unmanipulated contexts in sanctuary chimpanzees. This behavior‐based bottom‐up approach to the study of personality in chimpanzees can be useful in assessing consistency across naturally occurring diverse contexts. Most behaviors in this study were consistent across contexts (64%), half were consistent across time, and half fit with the personality structure typically found for chimpanzees (e.g., Koski, 2011; Massen et al., 2013; Uher et al., 2008). We, therefore, found some amount of contextual and temporal consistency in personality‐based behaviors of 22 sanctuary chimpanzees, observed reliably in their day‐to‐day life in multiple naturally occurring contexts, across a 4‐year time span.

More specifically, contextual consistency was found between contexts that differ on the functional, affective, arousal, and social level. Explorative behaviors were consistent across both solitary and social contexts, and sociability‐related behaviors were consistent across feeding, affiliation, and resting contexts. Personality‐based behavioral differences found across naturally occurring contexts have been rarely measured. Although past experimental studies contributed greatly to the field by demonstrating that individual nonhuman primates can be consistent in their personality‐based behaviors across multiple situations (e.g., Massen et al., 2013; Šlipogor et al., 2016), these studies examined the consistency of personality traits across very specific situations (e.g., small vs big novel objects to measure exploration‐avoidance: Šlipogor et al., 2016) and were arguably similar to the extent that they could be categorized as belonging to the same singular context.

Past research that examined contextual and temporal consistency revealed consistency for nonhuman primate personality and was observed for a range of personality traits, including boldness, explorativeness, anxiety, and sociability (e.g., Koski, 2011; Kutsukake et al., 2012; Massen et al., 2013). In our study, most of the behaviors were consistent across contexts and time, but one behavior (Groom) only showed consistency across contexts but not over time. One study in wild adult chimpanzees reported long‐term consistent differences in their social behaviors (Tkaczynski et al., 2020). However, the authors used a different approach to determine the consistency of the subjects' social phenotype. Here, by determining the real‐life contexts based on the activity of the conspecifics, we may have provided more detailed information about the consistency of the subjects' social behaviors, which can be adjusted according to the social environment (Mielke et al., 2018). The different levels of consistency among the behaviors may be explained in that the contexts and/or time periods affect the expression of the personality‐linked behaviors because of possible event occurrences, such as changes in the social environment which can be easily and objectively quantified in nonhuman primates, allowing, thus, to see how consistency may change. Such effects may result in higher levels of consistency between some contexts over some periods of time as previously observed in an experimental study in common marmosets (Koski & Burkart, 2015).

Personality traits can be expressed by different behaviors in different contexts and either or both can vary throughout a lifetime, highlighting the complexity of measuring personality. For instance, sociability can be assessed in feeding, affiliative and resting contexts while targeting different behaviors (e.g., body contact, food sharing). By capturing the range of distinctive contexts, including the ones that elicit different occurrences of personality‐based behaviors, we are likely to target different facets of expression of the same personality trait (Carter et al., 2012), and therefore, fully understand the targeted personality trait. The use of personality‐based approaches, such as the one developed in this study, has the potential to benefit conservation programs and help better understand great apes in rehabilitation centers (Rocque et al., 2022; Russon, 2006). Specifically, by focusing on individual differences, wildlife authorities in rehabilitation centers could, along with other considerations for their decision‐making, better identify the best candidates to ensure more successful releases in the wild (Norman et al., 2021; Rocque et al., 2022).

Each of the three personality factors obtained in our study was composed of behaviors that were similar to personality traits found in previous chimpanzee personality studies that used experimental and/or behavioral observations (e.g., Freeman et al., 2013; Koski, 2011; Kutsukake et al., 2012; Massen et al., 2013; Uher et al., 2008). Explorativeness and anxiety were composed of behaviors that have been previously associated with similar personality traits when measured in an experimental (exploration‐persistence: Massen et al., 2013; curiosity: Uher et al., 2008), and natural setting (anxiety: Koski, 2011). To our knowledge, boldness has only been examined in specific experimental situations (e.g., predator‐like situations: Massen et al., 2013), making direct comparisons with our study more challenging. Sociability, in our study, was expressed in conjunction with two other traits: boldness and anxiety. As boldness and sociability‐related behaviors were only measured in social contexts, their social connotations may have drawn them together on the same factor. Regarding the anxiety‐sociability trait, Groom, beyond its social characteristic, may have played a different function as this behavior has been reported to alleviate stress levels of distressed conspecifics (Fraser et al., 2008), explaining why they might load onto the same factor in our study.

Different factors could influence personality consistency, such as the audience surrounding the focal subject. Chimpanzees are known to be able to adjust their behaviors or decisions according to their social surrounding when it comes to social play (Flack et al., 2004) or grooming (Mielke et al., 2018). Similar patterns may be observed with behaviors that reflect an individual's personality, as the subjects may be keen on adjusting their behaviors to increase their fitness. However, it is unlikely to have had an impact on our data as we found consistency across both social and solitary contexts for explorativeness (Gaze Touch and Gaze Manipulate) and anxiety‐related behaviors (Self‐scratch, Vigilant and Self‐touch), behaviors that seemed not to be influenced by social cues in our study. Further investigation is needed to determine whether sociability and boldness‐related behaviors can be affected by the social environment (e.g., presence of high‐ranked individuals).

Regarding sex and age effects, previous research reported conflicting outcomes. Some studies showed that males were more anxious, active, or dominant than females (Dutton, 2008; Koski, 2011; Pederson et al., 2005; Weiss & King, 2015) or found that younger chimpanzees were bolder than older individuals (Massen et al., 2013), whereas other studies did not find any effect of sex (Massen et al., 2013) or age (Herrmann et al., 2011). Our study revealed no effect of the sex or the age of the subjects on their personality scores.

Animal personality research has often considered personality‐based behaviors to be consistent (Gosling, 2008; Vonk et al., 2017), but we found a notable number of exceptions. We add a novel aspect to personality research, that is the consideration of individual consistency: four to six chimpanzees showed significant contextual consistency (depending on consideration of all contexts or only social contexts, respectively); and only three chimpanzees showed significant consistency in their behavioral profiles over 4 years. Although consistency is a key aspect of personality, ecologists investigating animal personality also revealed a certain flexible aspect of personality by developing an approach allowing researchers to jointly examine animal personality and individual plasticity within the same framework (see “behavioral reaction norms”: Dingemanse et al., 2010). Thus, individual primates may differ in the way they respond to the environment in which different patterns of temporal and contextual consistency in behaviors may be observed across individuals (Dingemanse & Wolf, 2013), which they benefit from fitness‐wise (Wolf & Weissing, 2012).

Our study revealed that anxiety‐sociability was expressed differently across enclosures, suggesting that the social environment might have influenced this personality trait. Such group differences have been previously reported for social‐related behaviors in chimpanzees (e.g., Davila‐Ross et al., 2011; van Leeuwen et al., 2018) and other personality traits, such as boldness and exploration in common marmosets (Koski & Burkart, 2015) or sociability, positive affect, equitability, and activity in chimpanzees (Koski, 2011). However, anxiety was not reported to differ across groups (Koski, 2011). The lack of consistency between our findings and other studies could be explained by the difference in the studied groups. Indeed, social group composition and rearing (Bard & Gardner, 1996; Koski & Burkart, 2015; Schuppli et al., 2017) as well as stages of rehabilitation (Damerius et al., 2017) or status within a social group (Foerster et al., 2016) in a captive/semicaptive or wild setting are factors that are likely to have an impact on an individual's behavior. These factors could result in variations in the expression of their personality traits over time and contexts, and in interindividual differences, contributing therefore to the expression of different profiles (Furr & Funder, 2004; Uher et al., 2008). Few nonhuman primate personality studies assess the consistency of both the behavior and the individual, that is, variable‐ and individual‐centered analyses (e.g., Suomi et al., 1996; Tomassetti et al., 2019; Uher et al., 2008). There were variable degrees of temporal and contextual consistency between the individuals reported in these studies, as found in our study. By focusing on both the variable (behavior) and individual (behavioral profile), we can better encompass the complexity of personality, in terms of how different individuals express their personality through their behaviors and how personality is expressed across context and time. Although no statistical analyses were conducted here, the individual‐centered analysis also revealed that adults/subadults from the same colony showed consistency in their behavioral profile, and more specifically, only males in our samples showed temporal consistency.

Behavioral observation as a method to study personality has benefits, such as its strong ecological validity (Freeman et al., 2011) and its possibility to cover a range of distinct contexts and multiple behaviors displayed over different periods. However, behavioral observation also has its drawbacks, such as obtaining a data set that is large enough for each subject to prevent important variations across individuals. We conducted 53 h of recording, yet had fewer behavioral observations compared to other personality primate studies (e.g., Eckardt et al., 2015; Neumann et al., 2013) which could have contributed to unstable results in our study. Indeed, the wide variation in the duration across contexts (e.g., affiliative, solitude) might explain the variability in consistency across individuals. Yet, the fact that some individuals were observed for short periods of time in some contexts could also reflect a characteristic of their personality, such as being less social. Whereas the findings presented in this study must be carefully considered due to the small data set, we can see a pattern where behaviors show both contextual and temporal consistency when using behavioral observations.

By measuring personality in the day‐to‐day life of the chimpanzees, we were able to target various personality‐based behaviors expressed in a range of distinctive contexts on multiple occasions; therefore, we were able to better capture the complexity of expression of a personality trait which can show consistency, or a lack of consistency, in the daily life of the subjects and reveal interindividual differences. As different methods (ratings, experiments, and behavioral observations) present different strengths and weaknesses, future studies could combine such a behavioral approach with ratings and/or experimental observations. In return, comparing different methods could help provide a more detailed description and an improved understanding of the personality structure of nonhuman primate species (Chotard, 2019). In using such an approach (i.e., behavioral observation), we can facilitate comparative studies, and thus highlight similarities and/or variations across different primate species and different settings.

## AUTHOR CONTRIBUTIONS

**Hélène Chotard**: Conceptualization (equal); data curation (lead); formal analysis (lead); funding acquisition (lead); investigation (lead); methodology (lead); project administration (lead); resources (lead); software (lead); validation (lead); visualization (lead); writing – original draft (lead); writing – review & editing (lead). **Kim A. Bard**: Conceptualization (supporting); data curation (supporting); formal analysis (supporting); funding acquisition (supporting); investigation (supporting); methodology (supporting); project administration (supporting); resources (supporting); software (supporting); supervision (supporting); validation (supporting); visualization (supporting); writing – original draft (supporting); writing – review & editing (supporting). **Jérôme Micheletta**: Conceptualization (supporting); data curation (supporting); formal analysis (supporting); funding acquisition (supporting); investigation (supporting); methodology (supporting); project administration (supporting); resources (supporting); software (supporting); supervision (supporting); validation (supporting); visualization (supporting); writing – original draft (supporting); writing – review & editing (supporting). **Marina Davila‐Ross**: Conceptualization (equal); data curation (supporting); formal analysis (supporting); funding acquisition (supporting); investigation (supporting); methodology (supporting); project administration (supporting); resources (supporting); software (supporting); supervision (lead); validation (supporting); visualization (supporting); writing – original draft (supporting); writing – review & editing (lead).

## CONFLICT OF INTEREST

The authors declare no conflict of interest.

## Supporting information

Supporting information.

## Data Availability

All data used for analyses are available upon request.
