# Supplementary material for: Testing for personality consistency across naturally occurring behavioral contexts in sanctuary chimpanzees (Pan troglodytes)
Source: Am J Primatol. 2022 Nov 17;85(1):e23451. doi: 10.1002/ajp.23451 (PMC10078319; doi:10.1002/ajp.23451)
Supplement: Supplementary file 1 — Supporting information. [file AJP-85-0-s001.docx]

**Supplementary Tables**

**Table S1.** **Subject representation and colony composition.** Overview of the number of subjects for each year of recording per age and sex group per colony. Infants were not included in the study. A total of four adults/subadult chimpanzees and one juvenile from the 2013 recording period died prior to the 2017 recording period. Four infants were reported, for the 2017 recording period, born in between the two recording periods. Adults/subadults were over 8 years old, juveniles were 4-8 years old, and infants were less than 4 years old.

| Age group | Sex | 2013 | 2017 |
| --- | --- | --- | --- |
| Adults/subadults | Females | 4 | 8 |
|  | Males | 11 | 14 |
| Juveniles | Females | 4 | 0 |
|  | Males | 3 | 0 |

**Table S2.** **Behaviors and their definitions associated with the four personality traits of interest.** The table also depicts examples of previous behavioral and personality studies on nonhuman primates that mentioned these different behaviors.

| Personality trait | Behavior | Definition | Studies |
| --- | --- | --- | --- |
| Sociability | Groom | The subject or conspecific looks through a conspecific or subject’s hair while picking at dirt or others with fingers or lips | (Eckardt et al., 2015; Neumann et al., 2013) |
|  | Playful contact | The subject or conspecific initiates social interaction with a conspecific or subject | (Koski, 2011; Suomi et al., 1996) |
|  | Food sharing | The subject or conspecific gives food to, takes food without any resistance from another conspecific or subject or tolerates a conspecific or subject to take food from mouth to mouth or hand to hand | (Silk et al., 2013) |
|  | Food begging | The subject or conspecific is requesting food using hands or approaching his/her face towards conspecific/subject | (Freeman et al., 2013) |
|  | Body contact | The subject or conspecific touches gently the conspecific or subject's body, presents his/her arm when approaching, embraces a conspecific or subject, presents hand or finger in conspecific or subject's mouth, the subject or conspecific's mouth enters in contact with other's mouth or with other's body part | (Ebenau et al., 2019; van Hooff, 1973) |
| Boldness | Rough action | The subject jumps on conspecific, usually on the back, hits a conspecific with hands/feet, bites a conspecific, pulls a conspecific towards him/her or pushes a conspecific away with either hands/feet, shakes an object in the direction of a conspecific, shakes a conspecific's limb or swings own limb towards a conspecific or stomps the ground with hands/feet in direction of a conspecific | (Clay et al., 2015; Freeman et al., 2013) |
|  | Chase | The subject follows a conspecific (walks in a direct manner or runs) while the conspecific moves away | (Clay et al., 2015; Freeman et al., 2013) |
|  | Display | The subject shows a tense posture while moving around in a perturbed manner | (Goodall, 1986; Nishida et al., 1999) |
|  | Risky action | The subject grabs edible food within arms’ reach from a bigger/same size conspecific who can see the subject or steals edible food from a bigger/same size conspecific who resists in some ways | (Nishida et al., 1999) |
|  | Throw | The subject throws objects towards another conspecific | (Goodall, 1986; Nishida et al., 1999) |
|  | Risky approach | The subject approaches while gazing at conspecific(s) which put him/her in social danger | (Dammhahn & Almeling, 2012; Koski & Burkart, 2015; Santillán-Doherty et al., 2010) |
| Explorativeness | Gaze | The subject clearly directs gaze towards an object (e.g., stick, leaf, rock, rope, inedible food) or conspecific(s) (who are involved in social interaction, eating or manipulating an object) for at least 2 seconds. Part of the subject’s face needs to be visible to be counted | (Forss et al., 2015; Schuppli et al., 2017) |
|  | Gaze Approach | The subject moves towards an object or conspecific(s) while focusing on it. Part of the subject’s face needs to be visible to be counted | (Massen et al., 2013; Santillán-Doherty et al., 2010) |
|  | Gaze Touch | The subject’s hand or foot enters into contact with an object or conspecific(s) while focusing on it. Part of the subject’s face needs to be visible to be counted | (Damerius et al., 2017; Uher et al., 2008) |
|  | Gaze Manipulate | The subject manipulates an object or joins/holds conspecifics (involved in social interactions) while focusing on it | (Damerius et al., 2017; Uher et al., 2008) |
| Anxiety | Yawning | The subject opens widely his/her mouth | (Baker & Aureli, 1997) |
|  | Self-scratch | The subject rakes his/her hair with fingernails including mainly fingers or hand' s movements (gentle scratch) or including large arm movements (rough-scratch) | (Aureli & Waal, 1997; Baker & Aureli, 1997) |
|  | Self-touch | The subject touches his/her face or body with hand | (Aureli & Waal, 1997; Baker & Aureli, 1997) |
|  | Escape | The subject leaves hurriedly | (Clay et al., 2015; Uher et al., 2008) |
|  | Vigilance | The subject approaches slowly conspecific, stops occasionally to look around, grabs some food hurriedly, keeps looking around and is jumpy or freezes with no movements or vocalizations and shows a tense posture | (Kalin & Shelton, 2003; Kutsukake, 2003) |
|  | Pilo-erection | The subject's hair stands up | (Nishida et al., 1999) |
|  | Rocking | The subject moves back and forth while sitting | (Clay et al., 2015; Uher et al., 2008) |

**Table S3.** **Definition of the analyzed contexts**. The right column of the table depicts the mean durations (minutes) of each context.

| **Context** | **Definition** | **Mean (SD)** |
| --- | --- | --- |
| Feeding | The conspecifics are eating food provided by the sanctuary or are foraging in the enclosure | 2.79 (1.92) |
| Play | The conspecifics are involved in a playful interaction | 1.02 (1.21) |
| Grooming | The conspecifics are looking through others’ hair | 1.67 (1.61) |
| Resting | The conspecifics are sitting, lying down and/or self-grooming | 1.78 (1.65) |
| Solitude | No conspecifics are present within 10 meters proximity | 2.61 (1.78) |
| Vigilance | The conspecifics are alert while paying attention to events (including food being prepared by keepers) happening inside or outside the enclosure (e.g., vocalizations in the background) | 1.13 1.39) |
| Aggression | The conspecifics are showing aggressive behaviors (including quarrels, fights, displays) | 0.25 (0.26) |
| Locomotion | The conspecifics are moving on the ground or in the trees | 0.68 (0.73) |

**Table S4**. **Context duration (minutes) per individual across the two years.** The context duration per individual per time period is shown in parenthesis (2013; 2017). Sum, percentage, mean and SD are calculated across all individuals for each context. *The context labelled as Other includes behaviors that rarely occurred in the data and did not belong elsewhere, such as object manipulation or copulation.

| ID | Feeding | Resting | Solitude | Grooming | Play | Vigilance | Locomotion | Aggression | Other* |
| --- | --- | --- | --- | --- | --- | --- | --- | --- | --- |
| 1 | 75.7 (32.6; 43.1) | 58.5 (22.5; 36.0) | 15.5 (10.3; 5.2) | 19.2 (9.3; 9.9) | 1.3 (0.9; 0.4) | 0.2 (0.2; 0.0) | 5.0 (2.7; 2.3) | 0.0 (0.0; 0.0) | 3.2 (0.0; 3.2) |
| 3 | 55.7 (8.0; 47.7) | 19.0 (5.9; 13.1) | 22.0 (3.7; 18.3) | 12.7 (1.9; 10.8) | 0.0 (0.0; 0.0) | 3.5 (0.0; 3.5) | 4.0 (0.3; 3.7) | 0.1 (0.0; 0.1) | 0.0 (0.0; 0.0) |
| 4 | 44.4 (20.0; 24.4) | 72.7 (36.9; 35.8) | 29.7 (8.9; 20.8) | 12.6 (4.2; 8.4) | 5.2 (4.7; 0.5) | 5.7 (0.4; 5.3) | 5.0 (3.1; 1.9) | 0.5 (0.0; 0.5) | 2.5 (0.0; 2.5) |
| 7 | 65.6 (23.3; 42.3) | 78.8 (31.4; 47.4) | 14.9 (11.0; 3.9) | 5.3 (2.2; 3.1) | 6.3 (6.3; 0.0) | 0.2 (0.2; 0.0) | 1.7 (0.3; 1.4) | 0.8 (0.1; 0.7) | 0.0 (0.0; 0.0) |
| 8 | 51.1 (10.7; 40.4) | 37.2 (4.0; 33.2) | 16.5 (4.0; 12.5) | 7.0 (0.0; 7.0) | 0.0 (0.0; 0.0) | 4.6 (0.0; 4.6) | 1.7 (0.0; 1.7) | 0.4 (0.00; 0.4) | 0.0 (0.0; 0.0) |
| 10 | 53.8 (4.0; 49.8) | 28.2 (11.8; 16.4) | 10.6 (0.0; 10.6) | 10.5 (2.7; 7.8) | 0.0 (0.0; 0.0) | 11.2 (0.0; 11.2) | 5.1 (1.1; 4.0) | 0.3 (0.00; 0.3) | 0.0 (0.0; 0.0) |
| 13 | 58.7 (8.0; 50.7) | 23.4 (8.4; 15.0) | 10.2 (0.0; 10.2) | 15.1 (5.1; 10.0) | 2.6 (2.2; 0.4) | 1.9 (0.0; 1.9) | 1.5 (0.0; 1.5) | 0.0 (0.0; 0.0) | 0.0 (0.0; 0.0) |
| 15 | 62.2 (9.0; 53.2) | 19.7 (1.3; 18.4) | 10.0 (4.1; 5.9) | 6.17 (0.00; 6.17) | 0.0 (0.0; 0.0) | 11.2 (0.0; 11.2) | 0.7 (0.7; 0.0) | 0.8 (0.0; 0.8) | 0.9 (0.9; 0.0) |
| 16 | 65.5 (4.0: 61.5) | 35.0 (15.6; 19.4) | 2.8 (0.0; 2.8) | 10.3 (0.0; 10.3) | 0.0 (0.0; 0.0) | 4.5 (0.0; 4.5) | 1.3 (0.4; 0.9) | 0.3 (0.0; 0.3) | 1.3 (1.3; 0.0) |
| 24 | 57.3 (3.3; 54.0) | 20.7 (0.0; 20.7) | 5.9 (2.0; 3.9) | 0.2 (0.2; 0.0) | 1.3 (0.0; 1.3) | 5.6 (0.4; 5.2) | 0.0 (0.0; 0.0) | 0.2 (0.0; 0.2) | 0.0 (0.0; 0.0) |
| 29 | 49.1 (21.9; 27.2) | 39.1 (15.5; 23.6) | 61.6 (28.4; 33.2) | 6.2 (5.6; 0.6) | 2.6 (2.1; 0.5) | 0.0 (0.0; 0.0) | 14.0 (0.3; 13.7) | 0.2 (0.0; 0.2) | 0.2 (0.0; 0.2) |
| 32 | 61.0 (16.0; 45.0) | 70.5 (42.1; 28.4) | 12.0 (2.0; 10.0) | 19.0 (7.3; 11.7) | 8.2 (4.3; 3.9) | 2.4 (1.6; 0.8) | 1.5 (1.3; 0.2) | 0.1 (0.1; 0.0) | 0.0 (0.0; 0.0) |
| 36 | 41.6 (4.0; 37.6) | 34.4 (8.0; 26.4) | 9.2 (1.1; 8.1) | 3.8 (0.0; 3.8) | 0.3 (0.0; 0.3) | 12.4 (2.9; 9.5) | 5.1 (0.0; 5.1) | 0.7 (0.0; 0.7) | 0.0 (0.0; 0.0) |
| 42 | 40.5 (7.4; 33.1) | 17.4 (10.8; 6.6) | 35.0 (0.0; 35.0) | 0.0 (0.0; 0.0) | 18.3 (0.9; 17.4) | 1.6 (0.0; 1.6) | 1.1 (0.0; 1.1) | 1.2 (0.1; 1.1) | 0.0 (0.0; 0.0) |
| 47 | 77.7 (27.1; 50.6) | 29.7 (26.7; 3.0) | 26.3 (8.9; 17.4) | 14.7 (3.3; 11.4) | 15.0 (10.0; 5.0) | 2.8 (2.7; 0.1) | 9.5 (0.0; 9.5) | 1.0 (0.0; 1.0) | 1.7 (0.0; 1.7) |
| 50 | 58.1 (15.4; 42.7) | 64.9 (27.1; 37.8) | 19.6 (6.1; 13.5) | 22.2 (13.2; 9.0) | 6.57 (6.42; 0.15) | 0.7 (0.4; 0.3) | 5.5 (4.0; 1.5) | 1.1 (1.1; 0.0) | 0.0 (0.0; 0.0) |
| 53 | 77.9 (14.3; 63.6) | 19.7 (7.7; 12.0) | 16.5 (4.0; 12.5) | 3.0 (0.0; 3.0) | 0.0 (0.0; 0.0) | 5.1 (0.0; 5.1) | 1.8 (1.0; 0.8) | 0.7 (0.0; 0.7) | 0.0 (0.0; 0.0) |
| 56 | 51.3 (3.6; 47.7) | 37.3 (15.1; 22.2) | 13.8 (0.0; 13.8) | 2.2 (2.2; 0.0) | 2.3 (2.3; 0.0) | 12.9 (0.8; 12.1) | 4.4 (2.5; 1.9) | 0.8 (0.0; 0.8) | 1.2 (0.9; 0.3) |
| 57 | 55.0 (0.0; 55.0) | 28.1 (7.8; 20.3) | 15.4 (3.0; 12.4) | 4.7 (0.7; 4.0) | 3.9 (3.3; 0.6) | 0.0 (0.0; 0.0) | 2.7 (0.2; 2.5) | 0.0 (0.0; 0.0) | 0.4 (0.0; 0.4) |
| 60 | 36.7 (6.7; 30.0) | 56.7 (21.2; 35.5) | 42.3 (28.1; 14.2) | 17.9 (8.4; 9.5) | 5.1 (3.8; 1.3) | 5.1 (5.1; 0.0) | 4.2 (2.4; 1.8) | 1.8 (0.5; 1.3) | 1.3 (0.0; 1.3) |
| 65 | 65.0 (8.0; 57.0) | 18.7 (0.8; 18.0) | 3.0 (0.0; 3.0) | 16.9 (2.9; 14.0) | 12.1 (11.8; 0.3) | 2.2 (0.0; 2.2) | 5.0 (0.0; 5.0) | 1.9 (0.0; 1.9) | 0.0 (0.0; 0.0) |
| 66 | 73.4 (36.7; 36.7) | 47.0 (21.3; 25.8) | 33.4 (7.8; 25.6) | 12.4 (5.0; 7.4) | 8.8 (7.1; 1.7) | 0.0 (0.0; 0.0) | 3.9 (2.6; 1.3) | 0.1 (0.0; 0.1) | 1.1 (0.0; 1.1) |
| Sum | 1277.2 (284.1; 993.1) | 856.7 (341.9; 514.8) | 426.1 (133.5; 292.6) | 221.9 (74.1; 147.8) | 99.9 (66.2; 33.7) | 93.8 (14.8; 79.0) | 84.4 (22.7; 61.7) | 12.9 (2.0; 10.9) | 13.8 (3.1; 10.7) |
| % | 41.4 (30.2; 46.3) | 27.8 (36.3; 24.0) | 13.8 (14.2; 13.7) | 7.2 (7.9; 6.9) | 3.2 (7.0; 1.6) | 3.0 (1.6; 3.7) | 2.7 (2.4; 2.9) | 0.4 (0.2; 0.5) | 0.5 (0.3; 0.5) |
| Mean | 58.1 (13.0; 45.1) | 38.9 (15.5; 23.4) | 19.4 (6.1; 13.3) | 10.1 (3.4; 6.7) | 4.5 (3.0; 1.5) | 4.3 (0.7; 3.6) | 3.8 (1.0; 2.8) | 0.6 (0.1; 0.5) | 0.6 (0.1; 0.5) |
| SD | 11.8 (10.1; 10.7) | 19.7 (11.8; 11.0) | 14.0 (8.0; 9.0) | 6.6 (3.6; 4.3) | 5.2 (3.5; 3.8) | 4.2 (1.3; 4.1) | 3.2 (1.2; 3.2) | 0.6 (0.3; 0.5) | 0.9 (0.4; 0.9) |

**Table S5. Individual-centered analysis of contextual and temporal consistency when considering only the social contexts (feeding, affiliative, resting).** The behavioral profile of each individual included 11 behaviors (Body contact, Groom, Playful contact, Rough action, Gaze, Gaze Approach, Gaze Touch, Gaze Manipulate, Self-scratch, Self-touch, Vigilant) and 17 behaviors (Groom, Playful contact, Food sharing, Food begging, Body contact, Rough action, Chase, Risky action, Gaze, Gaze Approach, Gaze Touch, Gaze Manipulate, Yawn, Self-scratch, Self-touch, Escape, Vigilant) for the contextual and temporal consistency analyses, respectively. All acceptable consistencies are in boldface.

|  | CONTEXT |  | TIME | | | | |
| --- | --- | --- | --- | --- | --- | --- | --- |
| ID | Cronbach's α |  | ICC(3,1) | 95% Confidence Interval | | F value | p value |
|  |  |  |  | Lower bound | Upper bound |  |  |
| 1 | 0.55 |  | 0.38 | -0.11 | 0.72 | 2.20 | 0.063 |
| 3 | 0.29 |  | -0.02 | -0.48 | 0.45 | 0.96 | 0.531 |
| 4 | **0.83** |  | 0.08 | -0.40 | 0.53 | 1.17 | 0.376 |
| 7 | 0.35 |  | -0.08 | -0.53 | 0.40 | 0.85 | 0.630 |
| 8 | -0.07 |  | 0.17 | -0.32 | 0.59 | 1.41 | 0.250 |
| 10 | -0.62 |  | 0.12 | -0.37 | 0.55 | 1.26 | 0.322 |
| 13 | 0.48 |  | 0.01 | -0.46 | 0.47 | 1.02 | 0.488 |
| 15 | 0.58 |  | -0.12 | -0.55 | 0.37 | 0.79 | 0.676 |
| 16 | -0.57 |  | 0.14 | -0.35 | 0.57 | 1.33 | 0.289 |
| 24 | -0.53 |  | 0.30 | -0.20 | 0.67 | 1.84 | 0.117 |
| 29 | **0.71** |  | **0.55** | 0.11 | 0.81 | 3.47 | 0.009 |
| 32 | **0.79** |  | 0.29 | -0.21 | 0.67 | 1.80 | 0.125 |
| 36 | -0.24 |  | -0.02 | -0.49 | 0.45 | 0.95 | 0.538 |
| 42 | 0.02 |  | -0.07 | -0.52 | 0.41 | 0.86 | 0.614 |
| 47 | 0.16 |  | **0.45** | -0.03 | 0.76 | 2.61 | 0.032 |
| 50 | **0.82** |  | **0.53** | 0.08 | 0.80 | 3.22 | 0.013 |
| 53 | 0.14 |  | -0.12 | -0.55 | 0.37 | 0.79 | 0.677 |
| 56 | -0.28 |  | -0.22 | -0.62 | 0.28 | 0.64 | 0.808 |
| 57 | 0.58 |  | 0.19 | -0.30 | 0.61 | 1.48 | 0.220 |
| 60 | **0.60** |  | 0.37 | -0.12 | 0.71 | 2.15 | 0.068 |
| 65 | 0.45 |  | 0.15 | -0.34 | 0.58 | 1.35 | 0.276 |
| 66 | **0.65** |  | -0.23 | -0.63 | 0.27 | 0.63 | 0.817 |

**Table S6**. Overview of the individuals showing consistency (C) or no consistency (NC) in their behavioral profile across contexts and time. The table also depicts which enclosure the individuals are from, the sex and the age category.

| ID | Enclosure | Sex | Age category* | Contextual consistency (based on four contexts) | Contextual consistency (based on social contexts) | Temporal consistency (based on four contexts) | Temporal consistency (based on social contexts) |
| --- | --- | --- | --- | --- | --- | --- | --- |
| 1 | 4 | Male | Adult/Subadult | NC | NC | NC | NC |
| 3 | 1 | Male | Adult/Subadult | NC | NC | NC | NC |
| 4 | 4 | Female | Adult/Subadult | NC | C | NC | NC |
| 7 | 4 | Male | Adult/Subadult | NC | NC | NC | NC |
| 8 | 1 | Male | Adult/Subadult | NC | NC | NC | NC |
| 10 | 1 | Male | Adult/Subadult | NC | NC | NC | NC |
| 13 | 1 | Female | Adult/Subadult | NC | NC | NC | NC |
| 15 | 1 | Male | Adult/Subadult | NC | NC | NC | NC |
| 16 | 1 | Male | Adult/Subadult | NC | NC | NC | NC |
| 24 | 1 | Female | Juvenile | NC | NC | NC | NC |
| 29 | 4 | Male | Adult/Subadult | C | C | C | C |
| 32 | 4 | Male | Adult/Subadult | C | C | NC | NC |
| 36 | 1 | Female | Juvenile | NC | NC | NC | NC |
| 42 | 1 | Female | Adult/Subadult | NC | NC | NC | NC |
| 47 | 4 | Male | Adult/Subadult | NC | NC | NC | C |
| 50 | 4 | Male | Juvenile | NC | C | C | C |
| 53 | 1 | Male | Adult/Subadult | NC | NC | NC | NC |
| 56 | 1 | Male | Adult/Subadult | NC | NC | NC | NC |
| 57 | 1 | Female | Adult/Subadult | C | NC | NC | NC |
| 60 | 4 | Male | Adult/Subadult | NC | C | C | NC |
| 65 | 1 | Female | Adult/Subadult | NC | NC | NC | NC |
| 66 | 4 | Female | Adult/Subadult | C | C | NC | NC |

* Based on Median age calculated across 2013 and 2017.

**Table S7.** Test statistics for LMs testing the influence of sex, age and enclosure on the three personality traits. Significant results are indicated in boldface.

| Personality trait | Measure | Estimate | SE | t value | p value |
| --- | --- | --- | --- | --- | --- |
| Explorativeness | Intercept | -0.34 | 0.54 | -0.63 | 0.536 |
|  | Sex (male) | 0.28 | 0.50 | 0.57 | 0.578 |
|  | Age | 0.00 | 0.03 | -0.07 | 0.948 |
|  | Enclosure (Enclosure 4) | 0.46 | 0.47 | 0.99 | 0.338 |
| Boldness-Sociability | Intercept | 0.13 | 0.49 | 0.26 | 0.798 |
|  | Sex (male) | 0.05 | 0.45 | 0.12 | 0.907 |
|  | Age | -0.03 | 0.02 | -1.21 | 0.242 |
|  | Enclosure (Enclosure 4) | 0.80 | 0.43 | 1.89 | 0.075 |
| Anxiety-Sociability | Intercept | -0.66 | 0.35 | -1.91 | 0.073 |
|  | Sex (male) | -0.58 | 0.32 | -1.80 | 0.088 |
|  | Age | 0.02 | 0.02 | 1.23 | 0.233 |
|  | Enclosure (Enclosure 4) | 1.66 | 0.30 | 5.44 | **< 0.001** |

**Table S8.** Contextual consistency of the behaviors across feeding and resting contexts. All acceptable consistencies are in boldface.

| Behaviors | Cronbach's α |
| --- | --- |
| Body contact | **0.63** |
| Groom | 0.47 |
| Playful contact | -0.02 |
| Rough action | 0.33 |
| Gaze | 0.00 |
| Gaze Approach | -0.42 |
| Gaze Manipulate | **0.91** |
| Gaze Touch | 0.21 |
| Self-scratch | 0.21 |
| Self-touch | 0.34 |
| Vigilant | 0.37 |

**Table S9.** Temporal consistency of the behaviors when considering feeding and resting contexts. All acceptable consistencies are in boldface.

| Behaviors | ICC(3,1) | 95% Confidence interval | | F value | p-value |
| --- | --- | --- | --- | --- | --- |
|  |  | Lower bound | Upper bound |  |  |
| Body contact | -0.10 | -0.49 | 0.33 | 0.82 | 0.677 |
| Groom | 0.04 | -0.38 | 0.44 | 1.08 | 0.434 |
| Playful contact | -0.26 | -0.61 | 0.17 | 0.58 | 0.887 |
| Rough action | 0.32 | -0.11 | 0.65 | 1.93 | 0.070 |
| Gaze | -0.11 | -0.50 | 0.31 | 0.80 | 0.696 |
| Gaze Approach | -0.17 | -0.55 | 0.26 | 0.71 | 0.782 |
| Gaze Manipulate | **0.51** | 0.12 | 0.76 | 3.05 | 0.007 |
| Gaze Touch | **0.68** | 0.37 | 0.85 | 5.24 | 0.000 |
| Self-scratch | 0.08 | -0.35 | 0.47 | 1.17 | 0.364 |
| Self-touch | -0.07 | -0.47 | 0.36 | 0.87 | 0.619 |
| Vigilant | -0.02 | -0.43 | 0.39 | 0.95 | 0.542 |
| Chase | **0.47** | 0.07 | 0.74 | 2.78 | 0.012 |
| Escape | -0.08 | -0.48 | 0.34 | 0.85 | 0.643 |
| Yawn | 0.03 | -0.38 | 0.44 | 1.07 | 0.439 |
| Food begging | **0.54** | 0.17 | 0.78 | 3.37 | 0.004 |
| Food sharing | -0.11 | -0.50 | 0.32 | 0.81 | 0.685 |
| Risky action | -0.05 | -0.46 | 0.37 | 0.90 | 0.597 |

**Table S10.** **Individual-centered analysis of contextual and temporal consistency including feeding and resting contexts.** The behavioral profile of each individual included 11 behaviors (Groom, Playful contact, Body contact, Rough action, Gaze, Gaze Approach, Gaze Touch, Gaze Manipulate, Self-scratch, Self-touch, Vigilant) and 17 behaviors (Groom, Playful contact, Food sharing, Food begging, Body contact, Rough action, Chase, Risky action, Gaze, Gaze Approach, Gaze Touch, Gaze Manipulate, Yawn, Self-scratch, Self-touch, Escape, Vigilant) for the contextual and temporal consistency analyses, respectively. Following the criteria defined in the Method section, all Cronbach’s α and ICC values above 0.6 are in bold.

|  | CONTEXT |  | TIME | | | | |
| --- | --- | --- | --- | --- | --- | --- | --- |
| ID | Cronbach's α |  | ICC(3,1) | 95% Confidence interval | | F value | p value |
|  |  |  |  | Lower bound | Upper bound |  |  |
| 1 | -0.03 |  | -0.04 | -0.50 | 0.44 | 0.93 | 0.558 |
| 3 | **0.60** |  | -0.13 | -0.56 | 0.36 | 0.77 | 0.694 |
| 4 | **0.68** |  | -0.03 | -0.49 | 0.45 | 0.95 | 0.542 |
| 7 | -0.01 |  | -0.15 | -0.58 | 0.34 | 0.74 | 0.725 |
| 8 | 0.37 |  | 0.21 | -0.28 | 0.62 | 1.54 | 0.198 |
| 10 | -1.50 |  | -0.17 | -0.59 | 0.32 | 0.70 | 0.754 |
| 13 | 0.34 |  | -0.07 | -0.52 | 0.41 | 0.87 | 0.604 |
| 15 | **0.75** |  | -0.08 | -0.53 | 0.41 | 0.86 | 0.617 |
| 16 | -0.34 |  | 0.20 | -0.30 | 0.61 | 1.50 | 0.213 |
| 24 | -0.34 |  | 0.20 | -0.30 | 0.61 | 1.50 | 0.213 |
| 29 | **0.74** |  | **0.82** | 0.57 | 0.93 | 9.95 | 0.000 |
| 32 | 0.59 |  | 0.26 | -0.24 | 0.65 | 1.71 | 0.147 |
| 36 | 0.17 |  | -0.16 | -0.59 | 0.33 | 0.72 | 0.742 |
| 42 | -0.39 |  | 0.04 | -0.44 | 0.50 | 1.07 | 0.445 |
| 47 | -0.34 |  | 0.36 | -0.14 | 0.71 | 2.10 | 0.074 |
| 50 | 0.57 |  | 0.21 | -0.29 | 0.62 | 1.52 | 0.205 |
| 53 | 0.01 |  | -0.14 | -0.57 | 0.35 | 0.76 | 0.708 |
| 56 | 0.18 |  | -0.16 | -0.59 | 0.33 | 0.72 | 0.738 |
| 57 | **0.61** |  | 0.10 | -0.39 | 0.54 | 1.22 | 0.347 |
| 60 | 0.07 |  | 0.35 | -0.14 | 0.70 | 2.09 | 0.075 |
| 65 | -0.27 |  | 0.02 | -0.45 | 0.48 | 1.04 | 0.469 |
| 66 | **0.71** |  | -0.16 | -0.59 | 0.33 | 0.72 | 0.743 |

**Supplementary Figure**

**
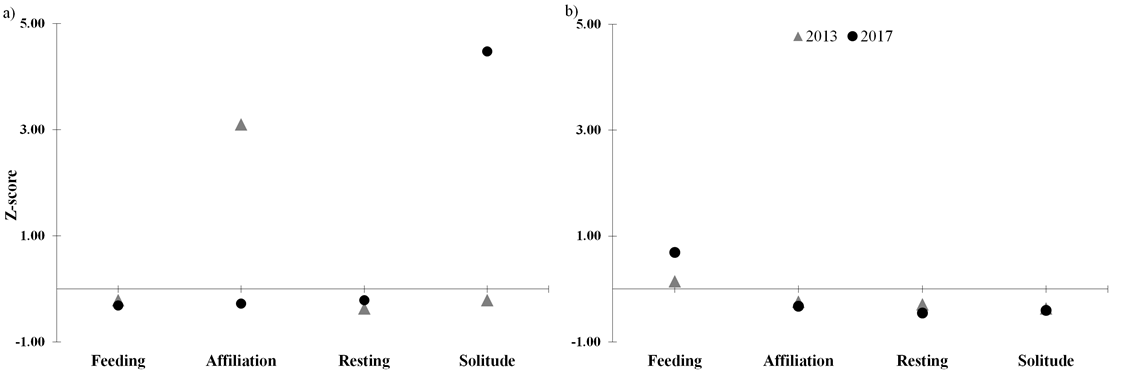
**

**Figure S1.** Examples of behaviors showing a) low (Gaze Approach) and b) high (Gaze Touch) contextual and temporal consistency. Figures 1a and 1b depict the z-score of the individual ID13 and the individual ID16, respectively. Here, the z-score reflects the score of the individual for one behavior in comparison with the other members of the group.

**Supplementary Results**

*Variable-centered approach*

*Feeding and resting contexts*

Seven out of 18 behaviors were not retained for the contextual analysis as they were expressed only in one of the two contexts (Food begging, Food sharing, Chase, Risky action, Escape, Rock, Yawn). To test for contextual consistency, the remaining 11 behaviors were combined over the two time periods. Here, only two behaviors show acceptable consistency (i.e., Cronbach’s α > 0.60) across feeding and resting contexts: Body contact and Gaze Manipulate (Table S8). The α values ranged from -0.42 to 0.47 for the other nine behaviors.

To test for temporal consistency, we combined feeding and resting contexts together. Here, we found temporal consistency for four behaviors: Gaze Manipulate, Gaze Touch, Chase and Food begging. The other 14 behaviors had low ICC values, which ranged from -0.26 to 0.32, indicating a lack of temporal consistency across the behaviors (Table S9).

*Individual-centered approach*

*Feeding and resting contexts*

When assessing the contextual consistency of the behavioral profile (11 behaviors displayed in both feeding and resting were included here), the individual-centered analysis revealed variability between the individuals. Six out of 22 individuals (27% of the sample) showed acceptable consistency across feeding and resting contexts (range: 0.60 – 0.75) whereas the other 16 individuals did not show any pattern of contextual consistency (see Table S10).

With regards to the temporal consistency of the behavioral profile (17 behaviors were included), the analysis revealed an excellent temporal consistency (ICC=0.82, p<0.05) in the pattern but only for one individual (5% of the sample). The other individuals did not show significant consistency in their behavioral profiles between 2013 and 2017 (see Table S10).
